# *Pneumocystis* Colonization in Dogs Is as in Humans

**DOI:** 10.3390/ijerph19063192

**Published:** 2022-03-08

**Authors:** Patrizia Danesi, Matteo Petini, Christian Falcaro, Michela Bertola, Elisa Mazzotta, Tommaso Furlanello, Mark Krockenberger, Richard Malik

**Affiliations:** 1 Parasitology, Mycology and Medical Enthomology, Istituto Zooprofilattico Sperimentale delle Venezie, Legnaro, 35020 Padua, Italy; cfalcaro@izsvenezie.it (C.F.); mbertola@izsvenezie.it (M.B.); emazzotta@izsvenezie.it (E.M.); 2 Clinica e Laboratorio Veterinario San Marco, Veggiano, 35030 Padua, Italy; matteo.petini@sanmarcovet.it (M.P.); tf@sanmarcovet.it (T.F.); 3 Veterinary Pathology Diagnostic Services, Sydney School of Veterinary Science, The University of Sydney, Sydney, NSW 2006, Australia; mark.krockenberger@sydney.edu.au; 4 Centre for Veterinary Education, The University of Sydney, Sydney, NSW 2006, Australia; richard.malik@sydney.edu.au

**Keywords:** *Pneumocystis canis*, dog, canine, pneumocystosis, Cavalier King Charles spaniel, pneumonia, bronchitis

## Abstract

*Pneumocystis* is an atypical fungus that resides in the pulmonary parenchyma of many mammals, including humans and dogs. Immunocompetent human hosts are usually asymptomatically colonised or show subtle clinical signs, but some immunocompromised people can develop florid life-threatening *Pneumocystis* pneumonia (PCP). Since much less is known concerning *Pneumocystis* in dogs, we posit the question: can *Pneumocystis* colonization be present in dogs with inflammatory airway or lung disease caused by other pathogens or disease processes? In this study, *Pneumocystis* DNA was detected in bronchoalveolar lavage fluid (BALF) of 22/255 dogs (9%) with respiratory distress and/or chronic cough. Although young dogs (<1 year-of-age) and pedigree breeds were more often *Pneumocystis*-qPCR positive than older dogs and crossbreds, adult dogs with other infectious conditions and/or a history of therapy-resistant pulmonary disease could also be qPCR-positive, including two patients with suppression of the immune system. Absence of pathognomonic clinical or radiographic signs render it impossible to convincingly discriminate between overt PCP *versus* other lung/airway disease processes colonised by *P. canis*. It is possible that colonisation with *P. canis* might play a certain role as a co-pathogen in some canine patients with lower respiratory disease.

## 1. Introduction

*Pneumocystis* is a genus of fungi with long evolutionary adaption to a commensal lifestyle in the pulmonary parenchyma of a wide range of mammals, including humans and dogs [1]. *Pneumocystis* can colonize individuals asymptomatically, but under conditions of impaired host immunity, it is capable of unchecked multiplication resulting in severe and life-threatening lung disease, commonly referred to as *Pneumocystis* pneumonia (PCP) or pneumocystosis [2].

*Pneumocystis* does not act as a zoonotic or anthropozoonotic pathogen, as each mammalian species studied thus far has its own host-adapted *Pneumocystis* species. Indeed, cross-infection experiments showed that *Pneumocystis* material obtained from a given animal species inoculated into a different (unrelated) animal species host is usually unable to induce infection and is rapidly eliminated from the lungs [1,3].

The taxonomy of *Pneumocystis* genus is continuously changing due to new and improved insights provided by phylogenetic studies. Five species have been described primarily in association with a specific host: *Pneumocystis jirovecii* in humans, *P. carinii* and *P. wakefieldiae* in brown rats (*Rattus norvegicus*), *P. murina* in mice (*Mus musculus*), and *P. oryctolagi* in rabbits (*Oryctolagus cuniculus*) [4,5,6]. *Pneumocystis canis* was recently described by whole genome sequencing as the cause of PCP in dogs. Interestingly, two distinct but very similar sequences have been amplified from a single Cavalier King Charles spaniel (CKCS) with PCP [7].

Animals may acquire the organism by (i) horizontal airborne transmission [8] from infected or asymptomatically colonized hosts; (ii) vertical transmission via the placental route, as suggested for rabbits and humans [9,10]; (iii) during the neonatal period, from parental infection/colonization [11,12]. Unlike the situation with *P. jirovecii* in humans, prevalence data for *Pneumocystis* in animal species is far less well documented. Canine cases of florid *Pneumocystis* pneumonia (PCP) have been fully described in only 53 dogs to date [13,14,15,16,17,18,19].

Although *Pneumocystis* is best known as a cause of severe disease in immunocompromised patients, the organism far more often acts as a transient or permanent commensal, colonizing limited portions of the lungs, while causing minimal or no damage to the host [20,21]. Thus, colonization and/or subclinical *Pneumocystis* infection may occur in immunocompetent dogs, cats and humans, including patients that have died of various causes unrelated to PCP carriage (such as vehicular trauma, chronic kidney disease or systemic viral illness) [22,23].

Symptomatic PCP in canine patients gives rise to variable and non-specific respiratory signs ranging from non-productive cough (not always present) to tachypnea and dyspnea with cyanosis, depending on the severity of the infection. Although a large variety of clinical and laboratory findings have been recorded, their diagnostic relevance remains limited, given that most findings are non-specific. It is important to state that the overall prevalence of symptomatic PCP in canine populations is very low, except in two pedigree populations viz. CKCS and miniature Dachshunds [24,25,26,27].

Establishing an antemortem diagnosis of PCP is not always straightforward. Definitive diagnosis bears two elements: (i) confirming presence of *Pneumocystis* using cytological or molecular methods, and (ii) deciding if it is the principal cause of lung disease in the patient, using a combination of history, physical and radiological findings, cytological assessment of a bronchoalveolar lavage fluid (BALF), molecular testing and response to anti-*Pneumocystis* drug therapy. To date, no study has looked at a representative cohort of canine patients with lower airway disease to determine how commonly *Pneumocystis* is present as an opportunistic pathogen, an incidental colonizer of abnormal lower airways, or absent.

The aim of this study was to investigate the prevalence of *Pneumocystis* in dogs and provide new information and discussion concerning the role of *Pneumocystis* in canine clinical respiratory disease. Over a three-year period, our molecular protocol for the diagnosis of *Pneumocystis* has established or confirmed the presence of *Pneumocystis* DNA in BALF from a cohort of 255 Italian dogs with disparate forms of lower respiratory tract disease. Real-time PCR enabled us to detect *Pneumocystis* DNA in BALF specimens from 22/255 dogs where *Pneumocystis* ‘cysts’ or ‘trophic forms’ were not detected cytologically. Critically, after considering all available clinical data, our conclusion was that in only three or less of the 22 dogs was PCP pneumonia a possible diagnosis, and that in most cases the presence of positive *P. canis* qPCR reflected colonisation of abnormal lung by *Pneumocystis* organisms. *Pneumocystis* might be contributing to disease features in some of these cases which occurred in variety of pedigree and crossbred dogs, including some with well understood causes of immune dysfunction, namely leishmaniosis and steroid responsive meningitis-arteritis (SRMA).

## 2. Materials and Methods

From 2017 to 2020, 255 canine BALF specimens were tested for presence of *Pneumocystis canis*. Samples were collected at the Clinica San Marco from dogs presented for veterinary attention because of lower respiratory disorders, mainly chronic cough and respiratory distress. Additional BALF specimen samples included in the study were sent to the Clinica San Marco for laboratory testing from other clinics. Signalment, histories and clinical findings are summarized in Table 1 and Table S1.

**Figure 1:**
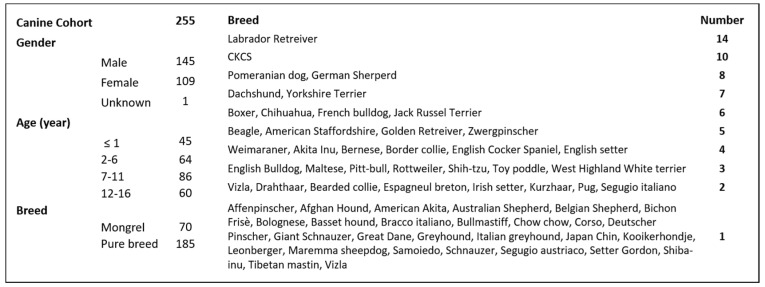
Details of overall canine cohort studied including list of breeds. Males and pedigree dogs were over-represented.

The cohort of dogs included in this study was composed of 70 crossbred dogs and 185 pedigree (purebred) dogs, with ages ranging from very young (<one-year-old) to 16 years and with males over-represented (Figure 1). Among the 64 different breeds and hybrids, Labrador retrievers and Cavalier King Charles Spaniels (CKCS) were most common, comprising 14 and 10 dogs, respectively; other breed(s) numbered less than 10 dogs each (Figure 1).

### 2.1. Molecular Testing

*Pneumocystis canis* molecular testing was performed at the Parasitology, Mycology and Medical entomology laboratory of the Istituto Zooprofilattico Sperimentale delle Venezie (IZSVe), Italy. At least 1 mL of BALF was tested for *Pneumocystis* DNA by Real-time polymerase chain reaction (qPCR) targeting a short fragment of the mitochondrial small subunit (150 bp). DNA extraction, SYBR Green Real-time PCR assay were performed using primers PneuSSU189/PneuSSU362 and conditions described previously [22]. All qPCR products that generated a single melting peak at 76 ± 0.5 °C, with C*_T_* values < 35 were considered to be positive [22].

### 2.2. Definition of PCP

In order for a patient to have *Pneumocystis* pneumonia (PCP), all the following criteria must be present: (i) a consistent signalment, history and physical findings, often including key breed associations (e.g., CKCS), possible immunosuppression and a history of chronic progressive dyspnoea/polypnoea with non-productive coughing (ii) chest radiographs demonstrating a heavy diffuse interstitial pattern (bordering on an alveolar pattern), often accompanied by right side heart enlargement (cor pulmonale) and echocardiographic evidence for pulmonary hypertension, with chest CT showing abundant ground glass opacities (iii) representative BALF specimen containing *Pneumocystis* ascii (cysts) or trophic forms (zoites) and/or qPCR positive for *P. canis* DNA with a C*_T_* below a defined cut-off, perhaps 26 (iv) unambiguous improvement in clinical status and radiological findings after treatment using trimethoprim sulpmethoxazole (often in concert with corticosteroids) and sometimes additional specific drugs directed against *Pneumocystis*.

### 2.3. Statistics

To identify groups of individuals with similar profile (i.e., gender, breed, age, *Pneumocystis* positive and co-infection) and the multiple correspondences between the levels of the categorical variables without any initial hypothesis, multiple correspondence analysis (MCA) were performed by using R Stats software, the FactoMineR for the analysis [28] and factoextra for data visualization [29] packages.

The differences in prevalence according to breed (crossbred vs. pure breed) and age (<1 year vs. older) were tested using two-tailed Fisher Exact tests, with 95% confidence limits. The software used was WinEpi (GraphPad Prism version 8.0.0 for Windows, GraphPad Software, San Diego, CA USA, available online at: https://www.graphpad.com accessed on 29 December 2021).

## 3. Results

### 3.1. Pneumocystis-Negative Dogs with Lower Airway and/or Lung Disease

In 233/255 (91%) dogs with lower respiratory tract disease, the qPCR for *P. canis* DNA was negative. The history, signalment, physical findings, diagnostic imaging and laboratory findings (including BALF cytology) for this large cohort of dogs with lung disease is presented in Table S1. None of these dogs, therefore, had clinical or subclinical PCP. Instead, a variety of disease conditions resulted in the clinical findings, including entities such as infectious ‘canine cough’, aspiration pneumonia and chronic bronchitis.

### 3.2. Pneumocystis-Positive Dogs with Lower Airway and Lung Disease with or without PCP

*Pneumocystis* DNA was detected in 22/255 dogs (8.8%) with lower airway or lung disease. Of the *P. canis*-qPCR positive dogs, 21 were purebreds, with a single crossbred dog. The positive pedigree breeds consisted of: Boxer dogs (*n* = 3; 14%), CKCS (*n* = 2; 9%), Pomeranian (*n* = 2; 9%) and one each of various other breeds (Figure 1). In this cohort, 16/22 (73%) of dogs were young (≤1 year old), while the remaining six dogs were 5 to 11 years old. There were 17 male dogs and five females.

To summarise the *P. canis* qPCR-positive cases from Table 1, but placed into conceptual categories, there was: CKCS with *Bordetella bronchiseptica, Mycoplasma* spp. and low Ct for *P. canis,* but no data on response to therapy (1), CKCS with *P. canis* with *Bordetella bronchiseptica* but no data on response to therapy (1), German Shepherd dog (GSD) with radiographs consistent with PCP, low Ct, ut no data on response to therapy (1), megaoesophagus but with imaging consistent with PCP (1), canine cough or chronic bronchitis due to *Bordetella bronchiseptica* and/or *Mycoplasma* spp. (11); eosinophilic bronchitis with *Bordetella bronchiseptica* and *Mycoplasma* (1), aspiration pneumonia (1); leishmaniasis (1); SRMA, possibly with immune-mediated vasculitis in the lung (1); *Mycoplasma* spp. infection treated with TMS (1), indeterminate (1). Cases 10, 11 and 13 was therefore tantalisingly close to a definitive diagnosis of PCP, yet some criterion was lacking, in many cases merely just due to poor follow-up, with no data on response to therapy.

Cough represented the most common clinical sign recorded. Thoracic radiographs were obtained for eight patients, and showed changes suggestive of PCP in 1/8 dogs, the remaining seven cases bearing radiographs with additional changes of bronchial disease and possible aspiration pneumonia or megaoesophagus. Computed tomography (CT) showed diffuse and/or multiple ‘ground glass’ opacities within the lung parenchyma in 7/10 dogs studied, with moderate enlargement of the tracheobronchial lymph nodes in 8/10 dogs.

BALF cytology was performed on specimens from 20/22 dogs. Neutrophilic inflammation (*n* = 9), granulomatous inflammation (*n* = 7) and septic inflammation (*n* = 1) were described. No *Pneumocystis* cysts or trophic forms were observed in stained smears of BALF. Bacterial infections were evident in 17/22 dogs, with *Bordetella bronchiseptica* (9) and/or *Mycoplasma pneumoniae* (8) co-infections confirmed using PCR testing in 12 canine BALFs.

Available haematological (*n* = 16) and biochemical (*n* = 14) measurements for the dogs showed that leukocytosis, hypoproteinaemia, and increased transaminases were the most common findings.

Treatment data were available for 11 dogs as reported in Table 1. Seven dogs improved. The dogs with megaoesophagus secondary to polyneuropathy died after 18 days and three other cases were lost to follow-up (Table 1). Importantly, only 3 dogs received TMS as a component of therapy.

In this cohort, *Pneumocystis* qPCR C*_T_* values ranged from 22 to 34. C*_T_* values lower than 26 were associated with three patients: a Bracco Italiano, a CKCS and a GSD. Taken together with the signalment, history, physical, radiological and laboratory findings, none of these dogs were considered to have unequivocal *Pneumocystis* pneumonia, although PCP was considered a potential diagnosis. Thus, in 19 dogs there were other likely causes of lower airway disease including bacterial pneumonia, chronic bronchitis, leishmaniasis and steroid responsive meningitis arteritis (SRMA), with the primary disease process complicated by colonisation with *P. canis*.

Thus, for patients with complete data available, a diagnosis of canine infectious respiratory disease (CIRD; also known as canine cough or kennel cough), bacterial pneumonia or chronic bronchitis was able to be confirmed in most cases. SRMA was diagnosed in one dog (the Bracco Italiano), while *Leishmania infantum* was diagnosed in an American Staffordshire bull terrier; in both these dogs, *P. canis* infection (PCP) was not considered the primary problem i.e., the cause of the dogs’ clinical signs. Indeed, the dog with SRMA was cured by immunosuppressive therapy alone, suggesting the lung changes detected with imaging were due to immune-mediated vascular disease in the lung parenchyma.

### 3.3. Comparison Positive/Negative Statistic Data

**Figure A1:**
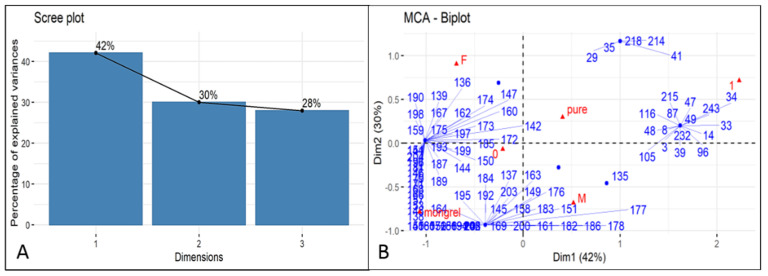
(**A**) Scree plot of the percentages of variance explained by each MCA dimensions (axes); (**B**) Biplot shows a global pattern within the data (individuals and variable categories): individuals are represented by blue points and variable categories by red triangles. 0 = *Pneumocystis* negative; 1 = *Pneumocystis* positive; pure = pure breed; mongrel= mixed breed; M = male; F = female. Number.

Among the 255 dogs, the multiple correspondence analysis (MCA) performed on ‘breed’, ‘gender’ and *Pneumocystis* variables, showed no specific association pattern (Figure A1).

Among the 22 *Pneumocystis* positive dogs, pedigree dogs were statistically more likely to be qPCR-positive for *Pneumocystis* than crossed breed (*p* = 0.0108), while young dogs (≤1 year old) were significantly more likely to be qPCR-positive than older dogs (*p* < 0.0001).

## 4. Discussion

In this study *Pneumocystis* DNA was present in 22/255 (9%) of BALF specimens from dogs with chronic respiratory distress or coughing. This compares to a previous study of ours, where 3/65 BALF samples from dogs with lower airway disease were *P. canis*-PCR positive with C*_T_* values of 27 to 31 [22]. Importantly, none of these dogs (from this and the previous study) revealed morphological evidence for *Pneumocystis* organisms (cysts or trophic forms) in stained smears of BALF. The extent to which *P. canis* contributes to disease in such dogs remains unknown. Indeed, it seems most likely that in a majority of cases the *P. canis* is not of clinical importance.

It is important, therefore, to emphasize that PCP cannot be diagnosed based on a positive qPCR result from a BALF specimen alone. To diagnose primary PCP, consistent clinical findings, characteristic changes in radiographs and CT scans (ideally high-resolution CT), and an unequivocal response to specific therapy with TMS, in addition to a positive qPCR result are required. In contradistinction, the presence of *Pneumocystis* cysts (ascii) or zoites (trophic forms) in stained smears of BALF is strongly associated with presence of symptomatic PCP; such cases are not included here but have been described previously [14,22] and are tabulated with the observed C*_T_* values for purposes of comparison to the cases presented in this study (Table 2).

C*_T_* values ranged from 22 to 34 in the 22 dogs in the qPCR-positive study cohort, consisting of dogs with disparate types of lung and airway disease. There remains a need to determine a robust cut-off C*_T_* value to help distinguish clinically meaningful presence of *Pneumocystis* DNA *versus* colonisation, most likely of no clinical importance. In the present study, the C*_T_* value was <26 in 3 dogs, a CKCS for which little clinical data was available (but which might have had PCP), a GSD with bacterial pneumonia due to *Acinetobacter berenziniae* and a Bracco Italiano with SRMA. Clearly, the presence of such C*_T_* values alone is insufficient to diagnose canine PCP. The major limitation of this study is that most of those dogs were lost in follow-up or had incomplete treatment response data, and the lack of treatment information does not permit a definitive diagnosis of PCP.

Currently, no guidelines exist in canine medicine to define if PCP is present in an individual patient. In the authors’ experience, to form a definitive diagnosis of PCP in a canine patient, some, ideally all, the following features need to be present:

- characteristic changes in chest radiographs, consisting of a dense and diffuse interstitial pattern, in association with right sided cardiac enlargement and signs of pulmonary hypertension on radiographs and using spectral Doppler echocardiography
- extensive ground glass densities in pulmonary CT scans
- the presence of cysts and/or trophozoite morphotypes on BALF cytology from stained smears (not always present but definitive when observed)
- *P. canis* qPCR positivity, with C*_T_* less than 26 (our current arbitrary cut-off)
- favourable response to TMS therapy, usually with corticosteroids for the first few days of therapy

In human patients, the diagnosis of PCP relies mainly on the detection of *P. jirovecii* by qPCR methods generally considered to be sensitive and specific, with the potential to distinguish colonization from infection using well validated cut-off values [30]. However, *P. jirovecii* load is affected by drug treatment(s) and underlying diseases, thus the interpretation of *P. jirovecii* infection by PCR is complicated by the need to discriminate between colonization and clinical disease. In a previous study, admittedly biased by the inclusion of many post-mortem specimens [22], a C*_T_* value lower than 26 was suggestive of infection by *P. canis* in dogs. In the current study, however, we are less certain that this cut-off value is accurate, as some dogs exhibited lower C*_T_* values despite apparently *not* having PCP.

To emphasize the complexity of interpretation of *P. jirovecii* PCR results in human patients, Yang and colleagues [31] showed that 9 BALF and 8 sputum samples from 17/70 patients without PCP were qPCR-positive with C*_T_* values > 30, whereas 14/51 BALF specimens and 20/70 sputum samples from patients with PCP were qPCR-negative. Clearly, the definitive diagnosis of PCP should be based on the full combination of features defined earlier.

An additional question relates to the role of *Pneumocystis* as an opportunistic pathogen. For example, in this study, the Bracco Italiano (Case 8, Table 1) recorded a low C*_T_* qPCR (<26), suggesting the lung disease might have in part been attributable to *Pneumocystis.* The dog was diagnosed with SRMA based on cerebrospinal fluid analysis and improved after immunosuppressive drug administration, but without TMS, suggesting SRMA as the principal cause of dog’s problems, including the lung disease. Indeed, *Pneumocystis* as an opportunistic pathogen can cause life-threatening pneumonia itself, but can also colonize the lungs of healthy hosts from a very early age, contributing to worsening of pulmonary function in cases with other underlying diseases [32,33].

In this study, amongst dogs with a qPCR-positive for *P. canis*, pedigree dogs and young dogs (*p* < 0.001) were at increased risk for PCR-positivity compared to crossbreds and older dogs, respectively. Interestingly, most young animals in this study were also co-infected with *Bordetella bronchiseptica* and/or *Mycoplasma* spp., suggesting infectious diseases are polymicrobial in young canine patients. Our suspicion is most of these dogs had infectious CIRD (also known as canine cough or kennel cough) and these patients were incidentally colonised by *P. canis*.

Among the Boxer dogs included in the overall study cohort (*n* = 6), three were qPCR-positive for *P. canis*. This is of potential importance as Boxer dogs are also overrepresented amongst cases of disseminated protothecosis and canine leproid granuloma syndrome [34,35]. Generally, there are two canine breeds well known to be at increased risk for the development of PCP, viz. the CKCS and the Miniature Dachshund. In the review by Weissenbacher-Lang et al. [13], 33/43 (77%) of dogs with symptomatic PCP were one of these two breeds, reflecting a likely predisposition due to congenital genetically-programmed immunodeficiency [27]. In relation to other affected breeds, certain lines of GSD can bear a predilection to developing disseminated fungal infections [36,37,38].

Distinct breeds of dogs with a predisposition for impaired innate or cell-mediated immunity are affected by PCP more often than dogs from immunologically competent breeds [13,26]. Nevertheless, immunodeficiency based or caused by co-infections or drug therapy (cyclosporine, corticosteroids, toceranib) is of equal importance for the acquisition of infections with *Pneumocystis*. Indeed, in this study, 12 dogs were co-infected with primary bacterial respiratory pathogens (*Bordetella bronchiseptica* and *Mycoplasma* spp.) and two dogs (the Bracco Italiano and the American Staffordshire bull terrier) had a concurrent steroid-responsive meningitis-arteritis (SRMA) and *Leishmania infantum* infection, respectively, both diseases capable of causing immune impairment.

The true significance and clinical consequences of *Pneumocystis* colonization are mostly unclear both in human and animals. *Pneumocystis* colonization might lead to acute PCP in susceptible hosts and/or result in transmission of the organism to others and/or stimulate pulmonary inflammation and lead to lung damage [33]. The immune response in a simian model infected with macaque-derived *Pneumocystis* showed that a cascade of cellular infiltration and mediator release in animals with acute PCP was similar to that seen in colonized animals that did not develop PCP. The intensity and the persistence of the inflammatory response observed in this model suggested the possibility that lung damage resulted from *Pneumocystis* colonization [39].

## 5. Conclusions

PCP represents a fatal disease in dogs with severe disease, which can be acute or acute on chronic. Real-time quantitative qPCR on BALF represents a promising and a relative ‘non-invasive’ method both to confirm colonisation or infection with *P. canis,* and to attest dogs are indeed negative for *P. canis.* This is especially the case when PCP is considered in the differential diagnosis but where specific staining methods, such as GMS for *Pneumocystis* cysts (ascii) in cytological preparations are negative. In this study, no cyst or trophic forms of *Pneumocystis* were evident in any cytological preparations, including the 22 cases that were qPCR positive. Factors including breed, age, evidence of impaired immunity, presence of chronic co-infections, as well as a history of long-standing therapy-resistant respiratory disorders are essential considerations for including pneumocystosis as a diagnostic possibility, or for making a presumptive clinical diagnosis. Beside the veterinary aim to improve canine PCP diagnosis, we need to bear in mind that dogs share the same urban and/or rural environment with humans. In the context of One Health medicine, animals with a particular genetic background living under similar conditions to humans might inform on disease pathogenesis and provide insights into the transmission and host-pathogen relationships in human disease.

## Figures and Tables

**Table 1:** Details of *Pneumocystis* positive dog including breed, gender, age, clinical signs, radiographic and laboratory findings, treatment, and outcome when available.

Case No.	Ct	Breed	Age (y)	Sex	Clinical Signs	Radiographs	CT	Cytology BAL	Co-Inf.	Bb (PCR)	My (PCR)	Other (Culture)	Diagnosis	Medical Treatment	Outcome
1	31	Akita Inu	1	ME	Fever, weakness, weight loss, tachypnoea	Right mid lobe consolidation with air bronchogram	‘Ground glass’ opacity of the cranial lobes. Moderate enlargement of the cranial mediastinal and sternal lymph nodes.	Pyo granulomatous septic inflammation	Yes	−ve	−ve	*Escherichia coli, Staphylococcus epidermis*	Pyo-granulomatous pneumonia	Amoxiclav and doxycycline	Alive after 7 months
2	30	American Stafford-shire	5	MN	Chronic cough	ND	Moderate ‘ground glass’ opacity of the peri-bronchovascular area. Slight enlargement of the cranial mediastinal and sternal lymph nodes.	Neutrophilic inflammation	Yes	−ve	−ve	Leishmania	Tracheo-bronchitis	Miltefosine and allopurinol	Alive after 11 months
3	30	Basset hound	<1	ME	Chronic cough	ND	ND	Mixed inflammation	Yes	+ve	−ve	ND	ND	ND	ND
4	29	Border collie	<1	FE	Chronic cough	ND	ND	Mixed inflammation	No	−ve	−ve	ND	ND	ND	ND
5	27	Boxer	<1	ME	Chronic cough	ND	ND	Neutrophilic inflammation	Yes	+ve	+ve	ND	ND	ND	ND
6	32	Boxer	<1	ME	Acute cough and respiratory dyspnoea	Diffuse broncho-interstitial-alveolar pattern	ND	Mixed inflammation	Yes	+ve	+ve	ND	Pneumonia	Doxycycline, TMS suggested but patient LTFU	LTFU
7	34	Boxer	<1	FE	Nasal discharge and chronic cough.	Moderate bronchial pattern	Multiple lung area of ‘ground glass’ opacity. Severe enlargement of the tracheobronchial lymph nodes.	Neutrophilic inflammation	Yes	+ve	+ve	*Pseudomonas* *aeruginosa*	Pneumonia	Amoxiclav and marbofloxacin	LTFU
8	25	Bracco Italiano	<1	ME	Joints pain, neck rigidity, chronic cough and fever.	ND	Diffuse ‘ground glass’ opacity of the left caudal lung lobe. Marked enlargement of the tracheobronchial lymph nodes.	Neutrophilic inflammation	No	−ve	−ve	ND	SRMA	Doxycycline, prednisolone, and cyclosporine (for SRMA)	Alive after 29 months
9	31	Chihuahua	<1	FE	Chronic cough	ND	Diffuse bronchial wall thickness	Septic inflammation	Yes	+ve	−ve	*Pseudomonas putida*	Septic Pneumonia and PCP	Doxycycline, marbofloxacin and TMS	Improved after one months, then LTFU
10	32	Chow Chow	<1	ME	Fever, weakness, and chronic cough	Diffuse bronchial-interstitial pattern	Diffuse bronchial wall thickness and diffuse ‘ground glass’ opacity of the lung. Enlargement of the sternal and tracheobronchial lymph nodes.	Neutrophilic inflammation	Yes	−ve	+ve	*Klebsiella pneumoniae*, *S. pseudointermedius*	Septic pneumonia and pulmonary pneumocystosis	TMS	Improved after 45 days
11	22	CKCS	<1	M	ND	ND	ND	Neutrophilic inflammation	Yes	+ve	+ve	ND	ND	ND	ND
12	34	CKCS	<1	ME	Chronic cough	ND	ND	Mixed inflammation	Yes	+ve	−ve	ND	Pneumonia	ND	ND
13	25	German Shepherd	6	ME	PU-PD and tachypnoea	ND	Disseminated ‘ground glass’ opacity. Enlargement and moderate enhancement of the tracheobronchial lymph nodes.	Mixed inflammation	Yes	−ve	−ve	*Acinetobacter berenziniae* (by BAL culture)	Pneumonia	Ceftriaxone	LTFU
14	28	Golden Retriever	11	ME	Acute cough	Diffuse oesophageal dilation, diffuse broncho-interstitial pattern	Diffuse oesophageal dilation, diffuse bilateral ‘ground glass’ opacity andperi bronchovascular thickening of various lung lobes.	Septic inflammation	Yes	−ve	+ve	*Enterobacter* *kobei*	Aspiration pneumonia due to mega-oesophagus secondary to polyneuropathy	Ceftriaxone	Dead after 18 days
15	32	Italian Grey-hound	8	ME	Cough and enforced respiratory sounds	Broncho-interstitial pattern of the right lung	Increased lung opacity of the peri-bronchovascular area of the cranial lung lobes. Slight enlargement of the tracheo-bronchial lymph nodes	Neutrophilic inflammation	Yes	−ve	+ve	NA	Pneumonia	Amoxyclav, doxycycline and TMS for amoxiclav	Improved
16	30	Labrador Retriever	<1	ME	Chronic cough	Bronchial pattern	ND	Septic inflammation	No	−ve	−ve	ND	ND	ND	ND
17	30	Mongrel	5	ME		ND	ND	ND	No	−ve	−ve	ND	ND	ND	ND
18	34	Pomeranian dog	<1	FN	Chronic cough	Normal	ND	Mixed inflammation	Yes	+ve	+ve	ND	Eosinophilic bronchitis	Fluticasone and doxycycline	Improved after 3 months, then LTFU
19	28	Pomeranian dog	11	MN	Chronic cough	ND	ND	Neutrophilic inflammation	No	−ve	−ve	ND	ND	ND	ND
20	30	Rottweiler	<1	FE	Cough	ND	ND	Neutrophilic inflammation	Yes	−ve	−ve	*Rhodococcus hoagii*	ND	ND	ND
21	31	Toy poodle	<1	ME	Chronic cough, acute vomiting and diarrhoea	ND	Normal lung and pseudoaneurysmal dilatation of the right auricle.	Normal	Yes	+ve	+ve	ND	Tracheo-bronchitis	NA	LYFU
22	27	Yorkshire Terrier	1	ME	Chronic cough	ND	ND	Septic inflammation	No	−ve	−ve	ND	ND	ND	ND

Ct = cycle threshold; ME = male entire; MN = male neutered; FE = female entire; CT = computed tomography; Co-inf. = co-infection; Bb = *Bordetella bronchiseptica*; My = *Mycoplasma* spp.; ND = not done; NA = not available; LTFU = lost to follow up; TMS = trimethoprim sulphonamide; Amoxyclav = amoxicillin clavulanate; PU-PD = polydipsia and polyuria.

**Table 2:** Canine specimens investigated with *Pneumocystis* qPCR. Comparison of C*_T_* values observed previously and in this study.

Classification	Specimens (No. of Cases)	Organisms	C*_T_*	Reference
Dogs with confirmed PCP	FFPE sections (3)	Numerous	<26	[22]
	DQ smears (2)	Numerous	<26	[22]
	BALF (3)	Numerous	<26	[22]
	BALF (1)	Numerous	<26	[14]
	DQ smears (2)	Occasional	27–34	[22]
Dogs with confirmed or suspected PCP	DQ (1)	None	27–34	[22]
	BALF (5)	None	27–34	[22]
	Lung tissue (1)	None	27–34	[22]
	DQ (3)	None	≥35	[22]
	BALF (62)	None	≥35	[22]
Dogs not suspected of having PCP	Lung tissue (10)	Not done	≥35	[22]
Dogs with LRT disease	BALF (19)	None	27–34	This study
Dogs colonised by *P. canis* with another cause of RT disease or with PCP	BALF (3)	None or Not done	<26	This study
Dogs with LRT disease but no colonisation by *P. canis*	BALF (233)	None or Not done	≥35	This study

FFPE = formalin-fixed paraffin-embedded tissue; DQ = Diff-Quik stain.

## Supplementary Materials

The following supporting information can be downloaded at: https://www.mdpi.com/article/10.3390/ijerph19063192/s1. Table S1: Details of PCP negative dogs (from 23 to 255) including breed, gender, age, clinical signs, radiographic and laboratory findings, treatment, and outcome when available.

## References

[1] Aliouat-Denis C.M., Chabé M., Demanche C., Aliouat E.M., Viscogliosi E., Guillot J., Delhaes L., Dei-Cas E. (2008). *Pneumocystis* species, co-evolution and pathogenic power. Infect. Genet. Evol..

[2] Dei-Cas E. (2000). *Pneumocystis* infections: The iceberg?. Med. Mycol..

[3] Durand-Joly I., Aliouat E.M., Recourt C., Guyot K., François N., Wauquier M., Camus D., Dei-Cas E. (2002). *Pneumocystis carinii* f. sp. *hominis* is not infectious for SCID mice. J. Clin. Microbiol..

[4] Frenkel J.K. (1976). *Pneumocystis jiroveci* n. sp. from man: Morphology, physiology, and immunology in relation to pathology. Natl. Cancer Inst. Monogr..

[5] Cushion M., Keely S., Stringer J. (2004). Molecular and phenotypic description of *Pneumocystis wakefieldiae* sp. nov., a new species in rats. Mycologia.

[6] Dei-Cas E., Chabé M., Moukhlis R., Durand-Joly I., Aliouat E.M., Stringer J.R., Cushion M., Noë C., Sybren De Hoog G., Guillot J. (2006). *Pneumocystis oryctolagi* sp. nov., an uncultured fungus causing pneumonia in rabbits at weaning: Review of current knowledge, and description of a new taxon on genotypic, phylogenetic and phenotypic bases. FEMS Microbiol. Rev..

[7] Cissé O.H., Ma L., Dekker J.P., Khil P.P., Youn J.H., Brenchley J.M., Blair R., Pahar B., Chabé M., Van Rompay K.K.A. (2021). Genomic insights into the host specific adaptation of the *Pneumocystis* genus. Commun. Biol..

[8] Aliouat-Denis C.M., Chabé M., Delhaes L., Dei-Cas E. (2014). Aerially transmitted human fungal pathogens: What can we learn from metagenomics and comparative genomics?. Rev. Iberoam. Micol..

[9] Chabé M., Aliouat E.M., Durand-Joly I., Gantois N., Conseil V., López C., Duriez T., Dei-Cas E., Vargas S.L. (2007). Exploring transplacental transmission of *Pneumocystis oryctolagi* in first-time pregnant and multiparous rabbit does. Med. Mycol..

[10] Montes-Cano M.A., Chabe M., Fontillon-Alberdi M., De La Horra C., Respaldiza N., Medrano F.J., Varela J.M., Dei-Cas E., Calderon E.J. (2009). Vertical transmission of *Pneumocystis jirovecii* in humans. Emerg. Infect. Dis..

[11] Vargas S.L., Hughes W.T., Santolaya M.E., Ulloa A.V., Ponce C.A., Cabrera C.E., Cumsille F., Gigliotti F. (2001). Search for primary infection by *Pneumocystis carinii* in a cohort of normal, healthy infants. Clin. Infect. Dis..

[12] Rojas P., Friaza V., García E., de la Horra C., Vargas S.L., Calderón E.J., Pavón A. (2017). Early Acquisition of *Pneumocystis jirovecii* colonization and potential association with respiratory distress syndrome in preterm newborn infants. Clin. Infect. Dis..

[13] Weissenbacher-Lang C., Fuchs-Baumgartinger A., Guija-De-Arespacochaga A., Klang A., Weissenböck H., Künzel F. (2018). Pneumocystosis in dogs: Meta-analysis of 43 published cases including clinical signs, diagnostic procedures, and treatment. J. Vet. Diagn. Investig..

[14] Best M.P., Boyd S.P., Danesi P. (2019). Confirmed case of *Pneumocystis* pneumonia in a Maltese Terrier × Papillon dog being treated with toceranib phosphate. Aust. Vet. J..

[15] Sakashita T., Kaneko Y., Izzati U.Z., Hirai T., Fuke N., Torisu S., Yamaguchi R. (2020). Disseminated pneumocystosis in a Toy Poodle. J. Comp. Pathol..

[16] Schiborra F., Scudder C.J., Littler R.M., Lamb C.R., McConnell J.F., Maddox T.W. (2018). CT findings in *Pneumocystis carinii* pneumonia in five dogs. Small Anim. Pract..

[17] Okine A.A.K., Chapman S., Hostutler R.A., Livingston R. (2018). Diagnosis of *Pneumocystis* pneumonia in a 2-year-old King Charles Cavalier Spaniel using the polymerase chain reaction. Vet. Clin. Pathol..

[18] Petini M., Furlanello T., Danesi P., Zoia A. (2019). Nested–polymerase chain reaction detection of *Pneumocystis carinii* f. sp. *canis* in a suspected immunocompromised Cavalier King Charles spaniel with multiple infections. SAGE Open Med. Case Rep..

[19] Merrill K., Coffey E., Furrow E., Masseau I., Rindt H., Reinero C. (2021). X-linked CD40 ligand deficiency in a 1-year-old male Shih Tzu with secondary *Pneumocystis* pneumonia. J. Vet. Intern. Med..

[20] Alanio A., Bretagne S. (2017). *Pneumocystis jirovecii* detection in asymptomatic patients: What does its natural history tell us?. F1000Research.

[21] Morris A., Norris K.A. (2012). Colonization by *Pneumocystis jirovecii* and its role in disease. Clin. Microbiol. Rev..

[22] Danesi P., Ravagnan S., Johnson L.R., Furlanello T., Milani A., Martin P., Boyd S., Best M., Galgut B., Irwin P. (2017). Molecular diagnosis of *Pneumocystis* pneumonia in dogs. Med. Mycol..

[23] Danesi P., Corrò M., Falcaro C., Carminato A., Furlanello T., Cocchi M., Krockenberger M.B., Meyer W., Capelli G., Malik R. (2019). Molecular detection of *Pneumocystis* in the lungs of cats. Med. Mycol..

[24] Ralph E., Reppas G., Halliday C., Krockenberger M., Malik R., Ralph E., Reppas G., Halliday C., Krockenberger M., Malik R. (2015). *Pneumocystis canis* pneumonia in dogs. Microbiol. Aust..

[25] Hagiwara Y., Fujiwara S., Takai H., Ohno K., Masuda K., Furuta T., Nakayama H., Doi K., Tsujimoto H. (2001). *Pneumocystis carinii* pneumonia in a Cavalier King Charles Spaniel. J. Vet. Med. Sci..

[26] Farrow B.R.H., Watson A.D.J., Hartley W.J., Huxtable C.R.R. (1972). *Pneumocystis* pneumonia in the dog. J. Comp. Pathol..

[27] Lobetti R. (2000). Common variable immunodeficiency in miniature dachshunds affected with *Pneumonocystis carinii* pneumonia. J. Vet. Diagn. Investig..

[28] Lê S., Josse J., Husson F. (2008). FactoMineR: An R Package for Multivariate Analysis. J. Stat. Softw..

[29] Kassambara A., Mundt F. Factoextra: Extract and Visualize the Results of Multivariate Data Analyses. https://CRAN.R-project.org/package=factoextra.

[30] Fauchier T., Hasseine L., Gari-Toussaint M., Casanova V., Marty P.M., Pomares C. (2016). Detection of *Pneumocystis jirovecii* by quantitative PCR to differentiate colonization and pneumonia in immunocompromised HIV-positive and HIV-negative patients. J. Clin. Microbiol..

[31] Yang S.L., Wen Y.H., Wu Y.S., Wang M.C., Chang P.Y., Yang S., Lu J.J. (2020). Diagnosis of *Pneumocystis* pneumonia by real-time PCR in patients with various underlying diseases. J. Microbiol. Immunol. Infect..

[32] Kureljušić B., Weissenbacher-Lang C., Nedorost N., Stixenberger D., Weissenböck H. (2016). Association between *Pneumocystis* spp. and co-infections with *Bordetella bronchiseptica*, *Mycoplasma hyopneumoniae* and *Pasteurella multocida* in Austrian pigs with pneumonia. Vet. J..

[33] Morris A., Wei K., Afshar K., Huang L. (2008). Epidemiology and clinical significance of *Pneumocystis* colonization. J. Infect. Dis..

[34] Stenner V.J., MacKay B., King T., Barrs V.R.D., Irwin P., Abraham L., Swift N., Langer N., Bernays M., Hampson E. (2007). Protothecosis in 17 Australian dogs and a review of the canine literature. Med. Mycol..

[35] Malik R., Martin P., Wigney D., Swan D., Sattler P.S., Cibilic D., Allen J., Mitchell D.H., Chen S.C.A., Hughes M.S. (2001). Treatment of canine leproid granuloma syndrome: Preliminary findings in seven dogs. Aust. Vet. J..

[36] Krockenberger M.B., Swinney G., Martin P., Rothwell T.R.L., Malik R. (2011). Sequential opportunistic infections in two German Shepherd dogs. Aust. Vet. J..

[37] Elad D. (2019). Disseminated canine mold infections. Vet. J..

[38] Watt P.R., Robins G.M., Galloway A.M., O’Boyle D.A. (1995). Disseminated opportunistic fungal disease in dogs: 10 cases (1982–1990). J. Am. Vet. Med. Assoc..

[39] Board K.F., Patil S., Lebedeva I., Capuano S., Trichel A.M., Murphey-Corb M., Rajakumar P.A., Flynn J.L., Haidaris C.G., Norris K.A. (2003). Experimental *Pneumocystis carinii* pneumonia in simian immunodeficiency virus-infected rhesus macaques. J. Infect. Dis..

