# Supplementary material for: Pneumocystis Colonization in Dogs Is as in Humans
Source: Int J Environ Res Public Health. 2022 Mar 8;19(6):3192. doi: 10.3390/ijerph19063192 (PMC8955813; doi:10.3390/ijerph19063192)
Supplement: Supplementary file 1 [file ijerph-19-03192-s001.zip › ijerph-1584830-supplementary.pdf]

N.	ID	Breed	Age (years)	Gender	Cytology BAL	Co-infection	Bb	My	CPIV	Bacterial Colture	Diagnosis
**23**	5365	Affenpinscher	4	M	Eosinophilic inflammation	Pos	nd	nd	nd	*Acinetobacter baumannii* complex	Not available
**24**	6572	Afghan Hound	5	F	Normal	Pos	Neg	Pos	Neg	Neg	Interstitial pneumopathy
**25**	48685	Akita Inu	1	M	Normal	Neg	Neg	Neg	Neg	Neg	Pyogranolomatous pneumonia
**26**	6294	Akita Inu	1	M	Eosinophilic inflammation	nd	nd	nd	nd	nd	Not available
**27**	6059	Akita Inu	<1	M	Macrophagic and lymphocytic inflammation	Pos	Pos	Pos	Neg	Citrobacter spp.	Septic pneumonia
**28**	6714	American Akita	1	M	Normal	Pos	Neg	Neg	Neg	*Enterobacter cloacae*	Lymphoplasmacellular rhinitis
**29**	7405	American Staffordshire	5	F	Macrophagic and neutrophilic inflammation	Neg	Neg	Neg	Neg	Neg	Pneumopathy
**30**	6530	American Staffordshire	12	F	Lypophagocytosis and macrophagic inflammation	Pos	Neg	Neg	Neg	*Pseudomonas stutzeri*	Lung carcinoma
**31**	5607	American Staffordshire	1	F	Normal	Neg	Neg	Neg	Neg	Neg	Systemic Toxoplasmosis
**32**	6298	American Staffordshire	13	F	Neutrophilic inflammation	nd	nd	nd	nd	nd	Not available
**33**	8503	Australian Shepherd	10	M	Macrophagic and neutrophilic inflammation	Pos	Neg	Neg	Neg	*Staphylococcus cohnii*	Septic bronchitis
**34**	7060	Beagle	6	M	Neutrophilic infllammation	nd	nd	nd	nd	nd	Not available
**35**	7654	Beagle	3	M	Normal	nd	nd	nd	nd	nd	Lymphoplasmacellular rhinitis

**36**	5912	Beagle	12	M	Septic inflammation	Pos	Neg	Neg	Neg	*Escherichia coli*	Septic pneumonia
**37**	53333	Beagle	6	M	Neutrophilic inflammation	nd	Neg	Neg	Neg	nd	Not available
**38**	8449	Beagle	13	F	Normal	nd	nd	nd	nd	nd	Not available
**39**	5679	Bearded collie	13	F	Septic inflammation	Pos	nd	nd	nd	*Citrobacter braakii; Microbacterium paraoxydans*	Septic Pneumonia
**40**	5757	Bearded collie	13	F	Neutrophilic inflammation	Pos	nd	nd	nd	*Acinetobacter baumannii* complex	Interstitial pneumopathy
**41**	7944	Belgian Shepherd	13	F	Neutrophilic inflammation	Neg	Neg	Neg	Neg	Neg	Pneumonia
**42**	7609	Bernese	2	M	Normal	nd	Neg	Neg	Neg	Neg	Bronchopathy
**43**	6442	Bernese	8	F	Lung sarcoma associated with septic inflammation	Pos	Neg	Neg	Neg	*Klebsiella pneumoniae*	Istiocytic sarcoma
**44**	7897	Bernese	4	M	Neutrophilic inflammation	Pos	Neg	Neg	nd	*Raoultella planticola*	Not available
**45**	5557	Bernese	6	F	Haemorrhage	Pos	Neg	Neg	Neg	*Citrobacter braakii*	Alveolar interstitial pnuemopathy
**46**	43265	Bichon Frisè	1	M	not available	Neg	nd	nd	nd	nd	Not available
**47**	5769	Bolognese	8	M	Neutrophilic inflammation	nd	nd	nd	nd	*Pseudomonas aureginosa*	Tracheal collapse
**48**	5302	Border collie	<1	F	Neutrophilic inflammation with strongyloides larvae	Pos	Neg	Neg	Neg	*Serratia marcescens*	Angiostrongylosis
**49**	6099	Border collie	4	M	Macrophagic inflammation	Pos	Neg	Neg	Neg	*Stenotrophomonas maltophilia*	Bronchopathy

**50**	6341	Border collie	2	M	Septic inflammation	nd	nd	nd	nd	nd	Not available
**51**	8615	Boxer	6	M	Not diagnostic	Pos	nd	nd	nd	*Acinetobacter baumannii; Crhyseobacterium indologenes*	Septic pleuritis with pyothorax
**52**	6202	Boxer	1	F	Septic inflammation	nd	nd	nd	nd	nd	not available
**53**	5968	Boxer	<1	F	Suppurative inflammation	nd	nd	nd	nd	nd	Not available
**54**	6732	Bullmastiff	6	F	Lymphoma	nd	nd	nd	nd	nd	Not available
**55**	6539	Chihuahua	2	M	Neutrophilic inflammation	nd	nd	nd	nd	nd	Pharyngeal stenosis
**56**	7682	Chihuahua	12	M	Neutrophilic inflammation	nd	nd	nd	nd	nd	Not available
**57**	6120	Chihuahua	7	F	Not diagnostic	Neg	nd	nd	nd	nd	Brachycephalic airway syndrome
**58**	7958	Chihuahua	13	M	Normal	Pos	Neg	Pos	Neg	*Serratia marcescens*	Interstitial Pneumopathy
**59**	7496	Chihuahua	10	F	Lymphocytic inflammation	nd	nd	nd	nd	nd	Not available
**60**	8261	CKCS	2	M	Normal	nd	nd	nd	nd	nd	Not available
**61**	7492	CKCS	6	M	Lymphocytic inflammation	Pos	Neg	Neg	Pos	Neg	Tracheal and bronchial collapse
**62**	46014	CKCS	<1	M	Neutrophilic inflammation and suspected bordetellosis	nd	nd	nd	nd	nd	Not available
**63**	5511	CKCS	6	F	Normal	nd	nd	nd	nd	nd	Brachycephalic airway syndrome

**64**	60214	CKCS	1	F	not available	nd	nd	nd	nd	nd	Not available
**65**	49804	CKCS	5	F	Eosinophilic inflammation	Neg	Neg	Neg	Neg	Neg	Tracheal collapse
**66**	40918	CKCS	9	F	not available	nd	nd	nd	nd	nd	Not available
**67**	52124	CKCS	<1	M	nd	Pos	Neg	Pos	nd	Neg	Not available
**68**	7006	Corso	2	M	Eosinophilic inflammation	Pos	Neg	Neg	Neg	*Leclercia adecarboxylata*	Not available
**69**	6909	Dachshund	12	F	Neutrophilic inflammation	nd	nd	nd	nd	nd	Interstitial pneumopathy
**70**	57302	Dachshund	5	M	Neutrophilic inflammation	Pos	Neg	Pos	Neg	*Staphylococcus hominis*	Bacterial pneumonia
**71**	7484	Dachshund	11	M	Normal	nd	nd	nd	nd	nd	Not available
**72**	8439	Dachshund	5	F	Eosinophilic inflammation	Pos	Neg	Neg	Neg	*Acinetobacter iwoffii*	Bacterial bronchitis
**73**	51705	Dachshund	9	F	septic inflammation	Pos	Neg	Pos	Neg	*Neisseria zoodegmatis; Bergeyella zoohelcum*	Bacterial bronchitis
**74**	7427	Dachshund	6	M	Neutrophilic inflammation	Pos	Neg	Pos	Neg	*Escherichia coli*	Aspiration pneumonia
**75**	7435	Dachshund	12	M	Neutrophilic inflammation	Pos	Neg	Pos	Neg	Escherichia coli, staphylococcus pseudointermedius	Bacterial pneumonia
**76**	50969	Deutscher Pinscher	7	M	nd	nd	nd	nd	nd	nd	Not available
**77**	6258	Drahthaar	11	M	Septic inflammation	Pos	Neg	Pos	Neg	*Citrobacter werkmanii*	Aspiration pneumonia

**78**	8372	Drahthaar	<1	M	Normal	Neg	nd	nd	nd	nd	Not available
**79**	8454	English Bulldog	5	M	Not diagnostic	nd	nd	nd	nd	nd	Not available
**80**	8361	English Bulldog	11	M	Not diagnostic	nd	nd	nd	nd	nd	Not available
**81**	5777	English Bulldog	<1	M	Neutrophilic, lymphocytic and macrophagic inflammation	nd	nd	nd	nd	*Citrobacter werkmanii*	Brachycephalic airway syndrome
**82**	6605	English Cocker Spaniel	4	M	Macrophagic and neutrophilic inflammation	nd	nd	nd	nd	nd	Not available
**83**	7215	English Cocker Spaniel	13	M	Neutrophilic inflammation	nd	nd	nd	nd	nd	Not available
**84**	5526	English Cocker Spaniel	9		Not diagnostic	nd	nd	nd	nd	nd	Not available
**85**	7394	English Cocker Spaniel	11	M	Macrophagic and neutrophilic inflammation	Pos	Neg	Pos	Neg	Neg	Chronic bronchitis
**86**	6229	English setter	1	F	Not diagnostic	nd	nd	nd	nd	nd	Not available
**87**	7095	English setter	8	M	Not diagnostic	nd	nd	nd	nd	nd	Not available
**88**	6033	English setter	1	F	Septic inflammation	Pos	Neg	Neg	Neg	*Klebsiella oxytoca*	Bacterial pneumonia
**89**	6761 sx	English setter	6	M	Eosinophilic inflammation	nd	nd	nd	nd	nd	Not available
**90**	41590	Espagneul breton	12	F	nd	nd	nd	nd	nd	nd	Not available
**91**	7424	Espagneul breton	10	M	Eosinophilic and neutrophilic inflammation	Neg	Neg	Neg	Neg	Neg	Allergic pneumonia

**92**	6547	French bulldog	4	M	Eosinophilic inflammation	Pos	Neg	Neg	Neg	*Enterobacter cloacae*	Not available
**93**	6549	French bulldog	8	F	Not diagnostic	Pos	Neg	Neg	Neg	*Escherichia coli*	Aspiration pneumonia
**94**	6169	French bulldog	4	F	Normal	nd	nd	nd	nd	nd	Not available
**95**	6884	French bulldog	1	M	Neutrophilic inflammation	nd	nd	nd	nd	nd	Not available
**96**	6801	French bulldog	10	M	Septic inflammation	Pos	Neg	Neg	Neg	*Streptococcus canis*	Bacterial pneumonia
**97**	7136	French bulldog	3	F	Normal	Pos	Neg	Neg	Neg	*Enterobacter cloacae*	Brachycephalic airway syndrome
**98**	5883	German Sherperd	<1	F	Not diagnostic	Pos	Neg	Neg	Neg	*Acinetobacter baumannii* complex	Bronchopneumonia
**99**	7221	German Sherperd	13	M	Neutrophilic inflammation	Pos	Neg	Neg	Neg	*Escherichia coli*	Aspiration pneumonia
**100**	7604	German Sherperd	5	F	Neutrophilic and macrophagic inflammation with fungal elements suggestive of Aspergillus spp.	nd	nd	nd	nd	nd	Pulmonary aspergillosis
**101**	60375	German Sherperd	4	F	Neutrophilic inflammation	Pos	Neg	Neg	Neg	*Citrobacter braakii; Staphylococcus saprophyticus; Escherichia fergusonii; Candida guillermondii*	Not available
**102**	7310	German Sherperd	8	F	Neutrophilic inflammation	nd	nd	nd	nd	nd	Not available
**103**	7340	German Sherperd	<1	M	Neutrophilic inflammation	nd	nd	nd	nd	nd	Not available

**104**	6020	German Sherperd	10	M	Not diagnostic	nd	nd	nd	nd	nd	Not available
**105**	7185	Giant Schnauzer	10	M	Normal	nd	nd	nd	nd	nd	Bronchopathy
**106**	5573	Golden Retreiver	1	M	Neutrophilic Inflammation	nd	nd	nd	nd	nd	Not available
**107**	8573	Golden Retreiver	2	M	Not diagnostic	nd	nd	nd	nd	nd	Not available
**108**	60383	Golden Retreiver	8	M	nd	nd	nd	nd	nd	nd	Not available
**109**	7235	Golden Retreiver	2	M	Septic inflammation	Pos	Neg	Neg	Neg	*Klebisella pneumoniae, Enterococcus faecium*	Not available
**110**	5470	Great Dane	7	M	Septic inflammation	Pos	Neg	Neg	Neg	*Citrobacter werkmanii*	Bacterial pneumonia
**111**	8406	Greyhound	4	F	Neutrophilic inflammation	nd	nd	nd	nd	nd	Lung foreign body
**112**	7486	Irish setter	7	M	Neutrophilic inflammation	Pos	Neg	Neg	Pos	Neg	Pneumopathy
**113**	7404	Irish setter	13	M	Macrophagic and neutrophilic inflammation	Pos	nd	nd	nd	*Pseudomonas aeruginosa*	Bacterial bronchitis and laryngeal paralysis
**114**	7106	Jack Russel Terrier	6	F	Neutrophilic, macrophagic and lymphocytic inflammation	nd	nd	nd	nd	nd	Not available
**115**	55515	Jack Russel Terrier	2	F	Macrophagic and neutrophilic inflammation	Pos	Pos	Neg	Neg	Neg	Bacterial pneumonia
**116**	7597	Jack Russel Terrier	10	F	Normal	Pos	Pos	Neg	Neg	Neg	Bacterial pneumonia

**117**	52547	Jack Russel Terrier	6	F	septic inflammation	Pos	Pos	Neg	Neg	*Pseudomonas aeruginosa*	Bacterial pneumonia
**118**	6168	Jack Russel Terrier	6	M	Normal	nd	nd	nd	nd	nd	Not available
**119**	7468	Jack Russel Terrier	2	M	Neutrophilic inflammation	nd	nd	nd	nd	nd	Not available
**120**	8317	Japan Chin	10	F	Neutrophilic inflammation	Pos	Neg	Neg	Neg	*Staphylococcus pseudointermedius*	Chronich bronchitis and interstitial pneumopathy
**121**	7125	Kooikerhondje	8	F	Neutrophilic and lymphocytic inflammation	Pos	Neg	Neg	Neg	*Enterobacter cloacae*	Interstitial pneumopathy
**122**	5789	Kurzhaar	9	M	Neutrophilic-eosinophilic inflammation	Pos	Neg	Neg	Neg	*Citrobacter werkmanii*	Laryngeal paralysis
**123**	5991	Kurzhaar	2	M	Neutrophilic inflammation	nd	nd	nd	nd	nd	Not available
**124**	6869	Labrador Retreiver	13	M	Neutrophilic inflammation	Pos	Neg	Pos	Neg	Mycoplasma spp.	Chronic bronchitis and bronchomalacia
**125**	6384	Labrador Retreiver	2	M	Macrophagic and neutrophilic inflammation	nd	nd	nd	nd	nd	Not available
**126**	5684	Labrador Retreiver	8	F	Neutrophilic Inflammation	Pos	Neg	Neg	nd	*Citrobacter braakii*	Not available
**127**	8337	Labrador Retreiver	10	F	Strongylus larvae	Pos	Neg	Pos	Neg	Neg	Angiostrongylosis
**128**	5457	Labrador Retreiver	13	F	Suppurative inflammation	Pos	Pos	Pos	Neg	Neg	Bacterial pneumonia
**129**	5768	Labrador Retreiver	<1	F	Septic inflammation	Pos	Neg	Pos	Neg	*Pseudomonas stutzeri*	Bacterial pneumonia
**130**	7212	Labrador Retreiver	8	M	Neutrophilic inflammation	nd	nd	nd	nd	nd	Not available

**131**	7240	Labrador Retreiver	10	M	Lymphocytic inflammation	nd	nd	nd	nd	nd	Not available
**132**	6632	Labrador Retreiver	10	M	Neutrophilic, macrophagic and eosinophilic inflammation	nd	nd	nd	nd	nd	Not available
**133**	5331	Labrador Retreiver	10	M	Macrophagic and neutrophilic inflammation	Pos	Neg	Neg	Neg	*Stenotrophomonas maltophilia*	Not available
**134**	7797	Labrador Retreiver	12	M	Neutrophilic inflammation	Pos	Neg	Neg	Neg	*Pseudomona putida; Chryseobacterium indologenes*	Bacterial bronchitis
**135**	5973	Labrador Retreiver	13	F	Neutrophilic inflammation	nd	nd	nd	nd	nd	Not available
**136**	8334	Labrador Retreiver	10	F	Neutrophilic inflammation	nd	nd	nd	nd	nd	Not available
**137**	43250	Leonberger	8	M	Macrophagic and neutrophilic inflammation	Neg	Neg	Neg	Neg	*Enterobacter cloacae*	Lung tumor
**138**	7731	Maltese	6	M	Neutrophilic inflammation	nd	nd	nd	nd	nd	Not available
**139**	6771	Maltese	6	M	Normal	nd	nd	nd	nd	nd	Not available
**140**	6308	Maltese	10	F	Neutrophilic inflammation	nd	nd	nd	nd	nd	Not available
**141**	7671	Maremma sheepdog	8	M	Macrophagic and neutrophilic inflammation	nd	nd	nd	nd	nd	Not available
**142**	6964	Mongrel	8	F	Septic inflammation	Pos	Neg	Neg	Neg	*Escherichia coli*	Not available
**143**	6531	Mongrel	2	M	Not diagnostic	Pos	Neg	Pos	Neg	Neg	Uppur respiratory tract disease

**144**	6689	Mongrel	2	F	septic inflammation	Pos	Neg	Neg	Neg	*Enterobacter cloacae*	Bacterial pneumonia
**145**	7035	mongrel	8	F	Eosinophilic inflammation	Neg	Neg	Neg	Neg	Neg	Eosinophilic broncopneumonia
**146**	6303	Mongrel	13	M	Normal	nd	nd	nd	nd	nd	Chronic bronchitis
**147**	5969	Mongrel	8	F	Neutrophilic inflammation	Pos	Neg	Neg	Neg	*Stenotrophomonas maltophilia*	Aspiration pneumonia
**148**	51911	Mongrel	7	F	nd	nd	nd	nd	nd	nd	Not available
**149**	8371	Mongrel	14	F	septic inflammation	Pos	Neg	Neg	Neg	*Escherichia coli*	Not available
**150**	6315	Mongrel	12	F	Macrophagic and neutrophilic inflammation	Pos	Neg	Neg	Neg	*Pseudomonas stutzeri*	Bacterial pneumonia
**151**	5760	Mongrel	12	M	Neutrophilic inflammation	Neg	Neg	Neg	Neg	Neg	Lung lobe torsion plus interstitial pneumopathy
**152**	5980	Mongrel	4	F	Normal	nd	nd	nd	nd	nd	Not available
**153**	6113	Mongrel	13	F	Neutrophilic inflammation and haemorrhage	Pos	Neg	Neg	Neg	Acinetobacter baumannii complex	Bacterial pneumonia
**154**	5626	Mongrel	12	M	Neutrophilic Inflammation	nd	nd	nd	nd	nd	Chronic bronchitis
**155**	7325	Mongrel	13	M	Neutrophilic inflammation	Pos	Neg	Neg	Neg	Acinetobacter baumannii complex	Broncopneumopathy
**156**	6121	Mongrel	10	F	Macrophagic and neutrophilic inflammation	nd	nd	nd	nd	nd	Not available
**157**	6706	Mongrel	3	M	Neutrophilic inflammation	Pos	Neg	Neg	Neg	*Enterobacter cloacae*	Pneumonia

**158**	6134	Mongrel	13	M	Neoplasia	Neg	Neg	Neg	Neg	*Pseudomonas stutzeri*	Lung carcinoma
**159**	8327	mongrel	1	F	Eosinophilic, lymphocytic, macrophagic and neutrophilic inflammation	nd	nd	nd	nd	nd	Not available
**160**	57917	Mongrel	11	F	Macrophagic inflammation	Pos	Neg	Neg	Neg	*Stenotrophomonas maltophilia*	Not available
**161**	6190	Mongrel	4	M	septic inflammation	Pos	Neg	Neg	Neg	Acinetobacter spp.	Bacterial pneumonia
**162**	6642	Mongrel	8	M	Erythrophagocytosis	nd	nd	nd	nd	neg	Suspected lung neoplasia
**163**	6488	Mongrel	8	F	Neutrophilic inflammation	Pos	Neg	Pos	Neg	Acinetobacter baumannii complex	Chronic bronchitis
**164**	5584	Mongrel	15	M	Eosinophilic, macrophagic and neutrophilic inflammation	Pos	Neg	Neg	Neg	*Citrobacter braakii*	Chronic bronchitis
**165**	5900	Mongrel	5	F	Eosinophilic inflammation	Pos	Neg	pos	Neg	Mycoplasma spp.	Canine mycoplasmosis
**166**	41007	Mongrel	4	F	Eosinophilic inflammation	Pos	Neg	Neg	Neg	*Stenotrophomonas maltophilia*	Eosinophilic bronchitis
**167**	7253	Mongrel	2	M	Macrophagic and neutrophilic inflammation	Neg	Neg	Neg	Neg	Neg	Sterile pannicolitis and lymphadenitis
**168**	5687	Mongrel	8	F	Eosinophilic inflammation	nd	nd	nd	nd	nd	Not available
**169**	8003	Mongrel	12	M	Normal	nd	nd	nd	nd	nd	Not available
**170**	7332	Mongrel	13	M	Normal	nd	nd	nd	nd	nd	Laryngeal paralysis

**171**	5591	Mongrel	13	M	Macrophagic inflammation	Pos	Neg	Neg	Neg	*Stenotrophomonas maltophilia*	Not available
**172**	7472	Mongrel	11	F	Macrophagic and neutrophilic inflammation	nd	nd	nd	nd	nd	Not available
**173**	5974	Mongrel	10	F	Carcinoma	Pos	Neg	Neg	Neg	*Citrobacter freundii; Stenotrophomonas maltophilia*	Lung carcinoma
**174**	8257	Mongrel	15	F	Normal	Pos	Neg	Pos	Neg	Neg	Chronic bronchitis
**175**	5436	Mongrel	15	M	Lymphocytic and macrophagic inflammation	Pos	Neg	Pos	Neg	Neg	Mycoplasmosis
**176**	5746 A	Mongrel	14	M	Neutrophilic inflammation and haemorrhage	nd	nd	nd	nd	nd	Not available
**177**	5882	Mongrel	10	F	Septic inflammation	Pos	Neg	Pos	Neg	*Acinetobacter baumannii* complex	Aspiration pneumonia
**178**	6609	Mongrel	13	F	Neutrophilic inflammation	nd	nd	nd	nd	nd	Not available
**179**	7258	Mongrel	14	F	Normal	nd	nd	nd	nd	nd	Not available
**180**	5332	Mongrel	13	F	nd	nd	nd	nd	nd	nd	Not available
**181**	7832	Mongrel	10	F	Lymphocytic, macrophagic and neutrophilic inflammation	Pos	Neg	Pos	Neg	*Pseudomonas putida; Streptococcus canis*	Bacterial broncopneumonia
**182**	5731 A	Mongrel	5	M	Eosinophilic and macrophagic inflammation	nd	nd	nd	nd	nd	Not available
**183**	6271	Mongrel	5	M	Septic inflammation	nd	nd	nd	nd	nd	Not available

**184**	6284	Mongrel	5	M	Not diagnostic	Pos	Neg	Neg	Neg	*Stenotrophomonas maltophilia*	Interstitial pneumopathy
**185**	5333	Mongrel	10	F	Normal	nd	nd	nd	nd	nd	not available
**186**	6361	mongrel	12	M	nd	nd	nd	nd	nd	nd	Not available
**187**	6797	Mongrel	1	F	Septic inflammation	Pos	Pos	neg	neg	*Acinetobacter iwoffii*	Bacterial pneumonia
**188**	7761	Mongrel	13	M	Normal	Pos	Neg	Neg	Neg	*Staphylococcus hominis*	Tracheal and bronchial collapse
**189**	5791	Mongrel	8	M	Eosinophilic inflammation	nd	nd	nd	nd	nd	Not available
**190**	8370	Mongrel	13	M	NOrmal	Pos	Neg	Pos	Neg	Neg	Broncomalacia
**191**	8038	Mongrel	5	F	Normal	nd	nd	nd	nd	nd	Not available
**192**	8227	Mongrel	11	M	Normal	nd	nd	nd	nd	nd	Not available
**193**	8418	Mongrel	8	F	Normal	nd	nd	nd	nd	nd	Not available
**194**	7755	Mongrel	12	M	Normal	nd	nd	nd	nd	nd	Not available
**195**	7738	Mongrel	7	F	Macrophagic inflammation	Pos	Neg	Neg	Neg	*Klebsiella pneumoniae*	Not available
**196**	6603	Mongrel	11	F	Neutrophilic inflammation	Pos	Neg	Pos	Neg	*Enterobacter cloacae*	Bronchial collapse
**197**	8284	Mongrel	13	M	Normal	Nd	nd	nd	nd	nd	Interstitial pneumopathy
**198**	8191	Mongrel	14	M	Neutrophilic inflammation	nd	nd	nd	nd	nd	Lymphoplasmacellular rhinitis and pneumopathy

**199**	7978	Mongrel	14	F	Neutrophilic and eosinophilic inflammation	nd	nd	nd	nd	nd	Not available
**200**	7252	Mongrel	9	M	Normal	nd	nd	nd	nd	nd	Not available
**201**	8558	Mongrel	4	M	Normal	nd	nd	nd	nd	nd	Septic pleuritis
**202**	8593	Mongrel	<1	F	septic inflammation	nd	nd	nd	nd	*Enterobacter hormaechei*	not available
**203**	5907	Mongrel	9	F	Macrophagic and neutrophilic inflammation	nd	nd	nd	nd	nd	Not available
**204**	5496	Mongrel	11	F	Eosinophilic inflammation	nd	nd	nd	nd	nd	Not available
**205**	6111	Mongrel	16	F	Carcinoma	nd	nd	nd	nd	nd	Lung carcinoma
**206**	8596	Mongrel	10	M	Neutrophilic inflammation	nd	nd	nd	nd	nd	Not available
**207**	8392	Mongrel	9	M	Neutrophilic inflammation	nd	nd	nd	nd	nd	Not available
**208**	6317	Mongrel	5	M	Neutrophilic inflammation	Neg	Neg	Neg	Neg	*Acinetobacter baumannii complex*	Tracheal collapse
**209**	5370	Mongrel	13	M	Eosinophilic inflammation	nd	nd	nd	nd	nd	Not available
**210**	5369	Mongrel	8	F	Macrophagic and neutrophilic inflammation	nd	nd	nd	nd	nd	Not available
**211**	6087	Pitt-bull	12	F	Macrophagic and neutrophilic inflammation	nd	nd	nd	nd	nd	Bronchopathy/bronchiectasia
**212**	5478	Pitt-bull	7	F	Not diagnostic	nd	nd	nd	nd	nd	Not available

**213**	6710	Pitt-bull	7	M	Lymphoma	nd	nd	nd	nd	nd	Not available
**214**	6941	Pomeranian dog	10	F	Neutrophilic infllammation	nd	nd	nd	nd	nd	Not available
**215**	7684	Pomeranian dog	3	M	Normal	Pos	Neg	Neg	Neg	*Pseudomonas aeruginosa; Staphylococcus pseudointermedius*	Bacterial pneumonia
**216**	5325	Pomeranian dog	1	M	Normal	nd	nd	nd	nd	nd	Not available
**217**	52758	Pomeranian dog	7	M	Normal	Pos	Neg	Neg	Neg	*Streptococcus agalactiae*	Tracheal collapse
**218**	5977	Pomeranian dog	11	F	Macrophagic and neutrophilic inflammation	nd	nd	nd	nd	nd	Not available
**219**	7301	Pomeranian dog	11	M	Neutrophilic inflammation	nd	nd	nd	nd	nd	Not available
**220**	7322	Pug	7	M	Normal	nd	nd	nd	nd	nd	Not available
**221**	8362	Pug	10	M	Normal	nd	nd	nd	nd	nd	Not available
**222**	8073	Rottweiler	12	M	Not diagnostic	Pos	Neg	Pos	Neg	*Pseudomonas aeruginosa*	Bacterial broncopneumonia
**223**	7128	Rottweiler	8	M	Neutrophilic inflammation	Pos	nd	nd	nd	*Klebsiella pneumoniae; Staphilococcus pseudointermedius*	Aspiration pneumonia
**224**	8404	Samoiedo	10	F	Macrophagic inflammation	nd	nd	nd	nd	Neg	Tracheal collapse
**225**	5927	Schnauzer	12	M	Macrophagic and neutrophilic inflammation	Pos	Neg	Neg	Neg	Citrobacter spp.	Bronchopathy

**226**	7318	Segugio austriaco	<1	F	Neutrophilic inflammation	Pos	Neg	Neg	Neg	*Bordetella bronchiseptica*	Bacterial pneumonia
**227**	6220	Segugio Italiano	1	M	Eosinophilic inflammation	Neg	Neg	Neg	Neg	Neg	Eosinophilic pneumonia
**228**	5930	Segugio Italiano	13	M	Macrophagic and neutrophilic inflammation	Pos	Neg	Neg	Neg	Citrobacter spp.	Not available
**229**	5766 A	Setter Gordon	5	M	Eosinophilic inflammation	nd	nd	nd	nd	nd	Not available
**230**	51586	shiba-inu	4	F	Eosinophilic inflammation	nd	nd	nd	nd	nd	Eosinophilic pneumonia
**231**	5966	Shih-tzu	13	M	Eosinophilic inflammation	nd	nd	nd	nd	nd	Not available
**232**	51363	Shih-tzu	13	F	Normal	Pos	Neg	Neg	Neg	Neg	Chronic bronchopathy
**233**	5553	Shih-tzu	9	F	Neutrophilic inflammation	Pos	nd	nd	nd	*Citrobacter freundii*	Brachycephalic airway syndrome
**234**	7487	Tibetan mastin	10	M	Macrophagic inflammation	Pos	Neg	Neg	Pos	*Enterobacter cancerogenous*	Bronchitis
**235**	6550	Toy poddle	15	F	Neutrophilic inflammation	Pos	neg	neg	Pos	*Streptococcus canis*	Pnuemonia
**236**	6016	Toy poddle	13	F	Lymphocytic and macrophagic inflammation	Pos	Neg	Neg	Neg	*Citrobacter freundii*	Tracheal collapse and bronchomalacia
**237**	8398	Vizla	2	M	Eosinophilic and neutrophilic inflammation	Pos	Neg	Neg	Neg	*Escherichia coli*	Bacterial pneumonia
**238**	7943	Weimaraner	13	F	Eosinophilic inflammation	Pos	Neg	Neg	Neg	*Serratia marcescens*	Not available
**239**	7292	Weimaraner	1	M	Normal	Neg	Neg	Neg	Neg	Neg	Bronchopneumonia

**240**	8459	Weimaraner	1	M	Macrophagic and neutrophilic inflammation with larvae of strongylus	Neg	Neg	Neg	nd	Neg	Angiostrongylosis
**241**	7403	Weimaraner	8	F	Macrophagic and neutrophilic inflammation	nd	nd	nd	nd	nd	Pneumopathy
**242**	42668	West Highland White terrier	11	M	nd	nd	nd	nd	nd	nd	Not available
**243**	7183	West Highland White terrier	10	F	Septic inflammation	Pos	nd	nd	nd	nd	Rhinitis and pneumopathy
**244**	52659	West Highland White terrier	13	M	Macrophagic and neutrophilic inflammation	nd	nd	nd	nd	nd	Interstitial pneumopathy
**245**	5616	Yorkshire Terrier	6	F	Normal	nd	nd	nd	nd	nd	Not available
**246**	7639	Yorkshire Terrier	14	F	Neutrophilic inflammation	nd	nd	nd	nd	nd	Not available
**247**	6928	Yorkshire Terrier	8	F	Normal	Pos	Neg	Neg	Neg	Klebsiella pneumoniae	Tracheal collapse
**248**	6342	Yorkshire Terrier	14	M	Septic inflammation	Pos	Neg	Neg	Neg	*Stenotrophomonas maltophilia*	Pneumonia
**249**	5318	Yorkshire Terrier	9	M	Normal	Pos	Neg	Neg	Neg	*Serratia marcescens*	Tracheal collapse
**250**	8095	Yorkshire Terrier	11	M	Normal	Neg	Neg	Neg	Neg	Neg	Chronic bronchopathy
**251**	6912	Zwergpinscher	12	M	Not diagnostic	Pos	Neg	Neg	Neg	*Stenotrophomonas maltophilia*	Interstitial pneumopathy
**252**	6881	Zwergpinscher	10	F	Macrophagic and neutrophilic inflammation	Pos	Pos	Neg	Neg	*Klebsiella pneumoniae*	Bacterial pneumonia
**253**	8148	Zwergpinscher	13	F	Neutrophilic inflammation	Pos	Neg	Neg	Neg	*Serratia marcescens*	Interstitial pneumopathy

**254**	7630	Zwergpinscher	8	M	Normal	Neg	Neg	Neg	Neg	Neg	Bronchial and tracheal collapse
**255**	5524	Zwergpinscher	10	F	Septic inflammation	Pos	Neg	Neg	Neg	*Citrobacter braakii; Microbacterium paraoxydans*	Septic Bronchitis

Table S1: Details of PCP negative dogs (from 23 to 255) including breed, gender, age, clinical signs, radiographic and laboratory findings, treatment, and outcome when available.

Bp = *Bordetella bronchiseptica*

My= mycoplasma

CPIV = Canine parainfluenza virus

nd= not done
